# Editorial: School Attendance and Problematic School Absenteeism in Youth

**DOI:** 10.3389/fpsyg.2020.602242

**Published:** 2020-11-06

**Authors:** Christopher A. Kearney, David Heyne, Carolina Gonzálvez

**Affiliations:** ^1^Department of Psychology, University of Nevada, Las Vegas, NV, United States; ^2^Institute of Psychology, Leiden University, Leiden, Netherlands; ^3^Department of Developmental Psychology and Didactics, Universidad de Alicante, San Vincente del Raspeig, Alicante, Spain

**Keywords:** school attendance, school absenteeism, truancy, school attendance problems, school dropout

Children who attend school regularly, and adolescents who complete high school, are more likely to experience better quality of life and achieve greater success at social, academic, occupational, and other aspects of functioning during their lifespan than youth who receive little education. School attendance is thus a key foundational competency for young people. Youth who do not attend school on a regular basis, or who prematurely leave school before graduation, are also at risk for myriad economic and related drawbacks in adulthood.

School attendance, absenteeism, and related constructs such as truancy and school dropout have been studied historically by professionals in many disciplines that include education, psychology, social work, medicine, nursing, sociology, and criminal justice, among others. These professionals have assembled a rich, if sometimes disparate, set of research findings on this population, as well as assessment and intervention strategies. The field continues to evolve toward common theories, constructs, and strategies to encompass all youth with school attendance problems.

School attendance and problematic school absenteeism are important areas of study particularly in education as well as clinical and health psychology. These constructs are important in education due to linkage to lower academic performance and achievement, lower reading and mathematics test scores, fewer literacy skills, grade retention, and dropout. These constructs are also important in clinical and health psychology due to linkage to psychiatric disorders (particularly anxiety and depression), social isolation, internalizing and externalizing behavior problems, involvement with the juvenile justice system, and long-term issues in adulthood that include psychiatric, occupational, and marital problems as well as economic deprivation. Indeed, school absenteeism and school dropout are often considered critical public health issues.

The primary goal of this Research Topic was to disseminate state-of-the-art theory and research and empirically supported practices relevant to mental health, school-based, and other professionals worldwide who address youth with school attendance problems. A secondary goal of this Research Topic was to enhance consensus among varied professionals regarding definition, classification, etiology, assessment, and intervention for school attendance problems that can be useful worldwide and that can serve as a foundation for future research and clinical work in this area. As such, multidisciplinary articles that can help bridge gaps in understanding and addressing this important population, and that have particular relevance to school districts, populate this special series.

Several articles in the Research Topic focus on issues regarding definition and classification of school attendance problems. Kearney et al. (a) and Kearney et al. (b) provide a review and critique of categorical and dimensional approaches to defining and conceptualizing school attendance problems with an eye toward reconciling these approaches. The researchers provide a roadmap for possible future avenues with respect to early warning systems, preventative and intervention strategies, and adaptations to future changes in education and technology, among other domains. The authors also provide a first glimpse of a multidimensional multi-tiered system of supports pyramid model as a potential mechanism for reconciliation in this complex and fragmented field.

Other articles in the Research Topic also outline particular challenges to, and suggestions for, defining and conceptualizing school attendance problems. Keppens et al. report a weak association between self-reported unauthorized school absenteeism and registered unauthorized school absenteeism, particularly for certain demographic, academic, and family groups in Flanders. The researchers note that reliable and valid identification and detection systems for school attendance problems must properly account for mismatches in data sources. Gentle-Genitty et al. recommend a change in how we frame student absenteeism to better leverage attendance data toward proactive student support. Many students are disproportionately affected and harmed by school absenteeism policies that are not effective. Instead, attendance-focused tracking and skills building can improve teacher engagement and foster a positive school environment to convey to students that their attendance is valued. Birioukov-Brant and Brant-Birioukov provide detailed feedback from Canadian educators about their personal challenges with respect to balancing exceptional circumstances (abuse, poverty, violence, mental health problems) faced by many of their students vis-à-vis rigid school attendance policies. Staff members see how attendance policies marginalize their students and are thus unwilling to fully comply with these mandates, often forming their own de facto policies. The study illustrates further the pitfalls of a one-size-fits-all approach to school attendance problems.

Several articles in the Research Topic also focus on contextual and risk factors that contribute to the emergence of school attendance problems. Contextual risk factors for school attendance problems are sometimes categorized across youth, parent, family, peer, school, and broader community domains. Several of these domains are represented throughout this special series and help advance the field by providing particularly nuanced findings.

Within the youth domain, Askeland et al. expand on the well-established relationship between depression and school attendance problems by examining these issues along a continuum, based on a large sample of Norwegian adolescents. The researchers find that symptoms of depression were evident even at low levels of school absences and that the relationship between depressive symptoms and school absences was partially mediated by sleep duration. These findings have important implications for preventative or Tier 1 strategies for this population. In addition, Fornander and Kearney (a) investigate internalizing symptoms in American youth across various absenteeism severity levels using ensemble and classification and regression tree analysis. Lack of enjoyment at school, a key depressive symptom, was predictive of several levels of absenteeism, and worry and fatigue were more evident across less severe and more severe absenteeism severity levels. These findings also have ramifications for universal and early screening for youth with potential school attendance problems.

Several articles in the Research Topic focus as well on parent and family variables that impact school attendance problems. Fornander and Kearney (b) examine family environment variables for American youth across different levels of absenteeism severity. Higher levels of absenteeism were more closely related to lower achievement orientation, active-recreational orientation, cohesion, and expressiveness. Interestingly, family conflict was elevated at 5% absenteeism but lower at 10% absenteeism, suggesting that some families may eventually become frustrated and disengaged from attempting to solve a child's school attendance problem. Gausel and Bourguignon also look at perceived family anger with respect to Norwegian students dropping out of school. The authors find that former students expect their family members to be angrier at them for leaving vocational education than general education. Family members may be especially upset about the possibility of the student being ill-prepared for an increasingly competitive labor market. Wang et al. delve even deeper into the nuances of parenting and family environment among Chinese families, finding intricate patterns related to school engagement, a key predictor of absenteeism. Maternal and paternal behaviors had an interactive effect; dual emotional warmth and behavioral guidance were related to stronger school engagement and dual harsh discipline was related to weaker school engagement. Student motivation toward mastery of academic material mediated these relationships. The researchers note that parents in China often deliberately convey different attitudes to their offspring but that a more positive and collaborative parenting approach may be best.

Other contextual risk factors for school attendance problems include peer-oriented variables. Delgado et al. examine cyberbullying profiles across maintaining variables of school attendance problems in Spanish adolescents, finding that anxiety-based variables were related to enhanced victimization, aggression, aggression-victimization, and observation behaviors. Bullying is closely related to school attendance problems and this study advances the field by extending results to the virtual world and by illustrating the need for expanded prevention efforts. In addition, Gonzálvez et al. evaluate the psychometric qualities of the Spanish version of the Child and Adolescent Social Adaptive Functioning Scale. Social functioning was found to be a protective factor against anxiety-based school refusal behaviors but not for pursuing tangible reinforcements outside the school setting. Both studies support the need to better understand peer influences on school attendance problems, an under researched domain in this population.

School-related factors are also examined in some articles in the Research Topic. Filippello et al. examine Italian student satisfaction and frustration at school vis-à-vis perceived teacher support. Student satisfaction was positively predicted by perceived teacher support and negatively predicted by perceived teacher psychological control, and student frustration was positively predicted by teacher perceived psychological control. These effects had impacts on student absences as well. The researchers note the importance of advocating supportive teaching practices such as paying attention to student needs and modifying dysfunctional instructional styles. Seçer and Ulaş find in Turkish students that academic resilience mediates a relationship between anxiety sensitivity and school attachment and partially mediates a relationship between social and adaptive functioning and school absenteeism and attachment. The researchers note the protective aspect of academic resilience and advocate for broadening holistic and causal models for school attachment and problematic school absenteeism.

Several articles in the Research Topic also focus on broader community issues, most notably those related to migrant and immigration status. Rosenthal et al. interview 11 parents of teenagers with school attendance problems in Paris and find that many confront systemic challenges following migration. These challenges include mistrust and disappointment in the inequalities and racism evident in schools and healthcare systems. These broader challenges affect family abilities to engage in cultural blending and cause parents to rethink their views of parenthood. Martin et al. likewise interview school personnel to illustrate the challenges of assimilating students of transcultural backgrounds into schools with a French universalist ideology. The researchers note some successes as school personnel navigate these challenges but also advocate for developing transcultural training for professionals working with students with such backgrounds who have school attendance problems.

Residential mobility is another broader community variable closely related to school attendance problems. Green et al. report that more than one-third of students in their American sample moved at least once in the past year. A greater number of moves led to less school connectedness and perceived academic ability as well as to more violence and harassment as a victim and as a perpetrator. The researchers note the value of strategies that can identify and support students who move at a young age in order to prevent student disengagement and promote attendance and academic success early in their life trajectory. Haugan and Myhr examine Norwegian adolescents and their families along four main groups that intersect poorly educated and well-educated families and non-intact and intact families. Students that did not complete secondary education were more likely to have a greater number of residential changes, but this effect was particularly evident among poorly educated, intact families and especially among poorly educated families *per se*. The researchers advocate for efforts to improve intergenerational educational mobility to promote stable and sustainable life situations for vulnerable families.

Several articles in the Research Topic also focus on intervention aspects for youth with school attendance problems. Maeda and Heyne report on a rapid return to school approach for Japanese youth with attendance problems that is implemented by school staff members. A significant percentage (72%) of intervention cases were classified as treatment responders and 89% of these cases demonstrated a return to school in 1 week. The researchers note the cost-effectiveness of this approach and the fact that it can bypass a student who is resistant to traditional clinical care. They also discuss the indications and contra-indications for using the approach. Lomholt et al. report on a feasibility study for Back2School, a manual-based, modular transdiagnostic cognitive behavioral intervention used with Danish youth with school attendance problems. This intervention is designed to address a wide array of behavioral issues in addition to school attendance problems. Initial outcomes of the feasibility study revealed a significant increase in school attendance and decrease in psychological symptoms, as well as a significant increase in youth and parent self-efficacy. Subsequent adaptations included greater school consultation, broader recruitment methods, and an increase in staffing by psychologists because of the time required to deliver the intervention. Heyne et al. provide a review of constructs measured following intervention for school refusal to inform guidelines for outcome measurement in future treatment studies. Many studies in this area have focused heavily on attendance as well as measures of emotional and behavioral symptoms, global functioning, self-efficacy, and diagnosis, among other aspects. The researchers provide guidelines for direction in this area that include accurate school attendance data, measures with strong psychometric properties, comparable measures across studies, uniform time-points for assessment, consistent outcome criteria, and multiple stakeholders that report on outcome intervention.

We are deeply grateful to all of the researchers and authors that contributed to this Research Topic. We are particularly delighted by diverse representation from so many different countries. The articles illustrate the substantial complexity of this population and the intense challenges faced by those who try to solve school attendance problems. The articles further demonstrate the need for input from multiple perspectives, disciplines, agencies, and parties to address the wide array of contextual risk factors endemic to school absenteeism. We hope this series of articles serves as a springboard for enhanced definition, classification, etiologic, assessment, and intervention frameworks for this critical public health issue.

## Author Contributions

All authors listed have made a substantial, direct and intellectual contribution to the work, and approved it for publication.

## Conflict of Interest

The authors declare that the research was conducted in the absence of any commercial or financial relationships that could be construed as a potential conflict of interest.

